# Insulin Treatment of Hypertriglyceridemia During Pregnancy

**DOI:** 10.3389/fphar.2021.785756

**Published:** 2022-01-19

**Authors:** De-cui Cheng, Yao Su, Feifei Li, Xianming Xu

**Affiliations:** Department of Obstetrics and Gynecology, Shanghai General Hospital, Shanghai, China

**Keywords:** insulin, hypertriacylglycerolemia, pregnancy, hypercholesterolemia, fetus

## Abstract

**Objective:** This study aims to investigate the efficiency of insulin on the reduction of gestational lipid profiles and try to propose a real-world approach to assist clinicians.

**Methods:** A retrospective, single-centered cohort study of 35 cases was conducted from October 2018 to July 2021 in Shanghai General Hospital. SPSS version 25.0 was performed to analyze the whole data. For continuous variables, a paired-sample *t* test was carried out on each variable to make a comparison between before and after treatment.

**Results:** The average pre-pregnancy TGs and TCs of these patients were about 3.96 ± 1.42 mmol/L and 4.78 ± 1.18 mmol/L, respectively. The maximum of TG before insulin treatment was up to 64.62 and TC 20.43 mmol/L, which decreased to 17.34 and 4.92 mmol/L after intervention of the insulin drip. TG was noticed to fall by 77% and 12.71% of TG, respectively. The difference of TG and TC between pre-treatment and post-treatment were statistically significant (*p* < 0.01), while this difference has not been found in the other laboratory tests reports. The outcomes of newborns and mothers with management of insulin were proven to be improved.

**Conclusion:** The use of insulin in the management of gestational hypertriglyceridemia is safe and efficient, and insulin may become a mainstream in the near future to mitigate serum TG and TC levels in the pregnancy period besides regulating the blood glucose level.

## Introduction

During normal pregnancy, the concentration of all lipoprotein increases physiologically, which seem to be originally hormonally mediated, and most of the healthy pregnant women can tolerate such changes and will not cause obvious adverse outcomes. A rare but life-threatening exception is hypertriglyceridemia (HTG)-induced acute pancreatitis; besides, numerous studies have demonstrated that this growth of serum lipid profile has potentially other complications including preeclampsia, gestational diabetes (GDM), large for gestational age (LGA), small for gestational age (SGA), macrosomia, intrahepatic cholestasis syndrome during pregnancy (ICP), and furtherly, cardiovascular disease and a greater likehood for HTG in the further future. (Shenhav et al., 2002; Ray et al., 2006; Lewington et al., 2007; Basaran, 2009; Berglund et al., 2012; Goldberg and Hegele, 2012). Unfortunately, up to now there still has been lack of established practical guidelines for clinicians to deal with such cases as currently absence of safety data of this period for most lowering-serum lipid medicine. Being in such a particular period—pregnancy period—with no safe access to other treatment options, patients in our institution were given insulin drip as the related literature demonstrated. (Jin et al., 2018; Hoff and Piechowski, 2021).

Treatment for severe gestational hypertriglyceridemia (GHTG) is a big challenge for clinicians since it is complicated by the thought of potential jeopardy of medications to fetus; still, it should be initiated immediately and aggressively in view of the potential risks brought by HTG. In term of treatments, it is mandatory and urgent to correct the underlying conditions, such as uncontrolled diabetes and obesity which can intensify the degree of HTG. Dietary restriction of high-fat food such as cakes and biscuits has long been the cornerstone of therapy since lipid-lowering agents in pregnancy have been only scarcely studied in pregnant women, and there is limited information regarding fetal effects (Rouhi-Boroujeni et al., 2017),but this measure is usually not sufficient and strong enough to control the severity of HTG. The diet with <20% of calories from fat and rich in omega-3 fatty acid, should be initiated immediately as it can decrease the concentration of chylomicrons (Goldberg and Hegele, 2012). Admission to hospital for intensive dietary control is imperative, provided that the patient is not be able to conduct strict low-fat dietary, and being fasting in conjunction with total parenteral nutrition (TPN) is advised if necessary.

Lipid-lowering agents are the first line for non-pregnant women but are not recommended in this period, given the considerations of the potential possible adverse effects of the fetus. Omega-3 fatty acids, as a kind of therapy of monotherapy, are noticed to able to decrease TG and safe for pregnancy but only breed limited benefits (Barrett et al., 2014). Niacin and fibrates are mainstreams in the management of non-pregnant HTG; however, little data are available from well-designed studies for pregnant women. Although no significant side effects have been observed in case reports of fibrates (gemfibrozil), which is classified as category C drugs by the Food and Drug Administration (FDA), and niacin supplementation in early and mid-pregnancy, they are still left to be controversial, and further research is required to assist their permission in this particular period (Berglund et al., 2012; Cao et al., 2018; Newman et al., 2019). Statins are a kind of β-hydroxyl-β-meglutarate monoacylcoa reductase inhibitor (HMG-CoA), whose efficacy to downgrade TC has been proven very well, being classified as Class X drugs by the FDA, are believed not to be teratogenic by plenty of scholars, but evidence-based medical evidence is insufficient; therefore, they are temporarily banned during pregnancy.

Another choice of medicine is heparin as it increases LPL levels by promoting the release of lipoprotein esterase from the surface of endothelial cells into circulation and accelerate fat hydrolysis and further lower levels of TG (Scherer et al., 2014). Heparin facilitates pancreatic microcirculation, and blood viscosity of patients is also noticed to be improved; in the meanwhile, its existence decreases the expression of endotoxin and proinflammatory factors, (Lu et al., 2010); thus, it should have become a promising choice until another research showed that it accelerates the transfer of LPL from blood to the liver, subsidizes depletion of LPL in circulation, introduces the content of chylomicrons, slows down the metabolic rate of TG, and finally brings into increased TG concentration (Scherer et al., 2014). Plasmapheresis can reduce serum TG levels effectively and quickly but raise concerns about its cost-effectiveness, complications, and staffing resources, and these concerns are more pronounced in the pregnancy period. Our study aims to investigate the efficiency of insulin on the reduction of gestational lipid profiles. In this literature, we highlight and collect data prototypical cases of gestational hypertriglyceridemia (GHTG), mostly severe types, and some related clinical treatments, with a purpose of proposing a real-world approach to assist clinicians.

## Methods

This is a retrospective, single-centered cohort study, conducted between October 2018 and July 2021 in Shanghai General Hospital, approved by the ethics boards of Shanghai General Hospital. All participants provided written informed consent, and this study was conducted in accordance with the Declaration of Helsinki. This research was funded by the Chinese Clinical Trial Registry (ChiCTR2000036575).

Information presented in this study was collected from pregnant women’s electronic medical records and prenatal care documentary book, which includes medical records of the entire gestation period, from the hospital’s medical registries for detailed information on hospital admission and blood investigations during pregnancy to postpartum.

Eligible criteria include the following: *1*) Triglyceride is above 9 mmol/L. *2*) Insulin therapy for gestational HTG. *3*) No chronic diseases before pregnancy. *4*) No other treatments but insulin to deal with HTG. *5*). 20 years old ≤ age ≤40 years old.

Based on the above criteria, 35 cases were enrolled in our research.

1400 patients were diagnosed with HTCG (TG ≥ 2.25 mmol/L) from the year of 2018 to 2021, and the number of people whose TG were above 9 mmol/L was 48. After excluding three who were receiving other treatments (fenofibrate and niacin), three whose age were above 40 years old, four who were complicated with other chronic diseases (diabetes I, chronic hypertension, chronic pancreatitis), two whose lipid levels improved a lot after diet control (from 10 to 8 mmol/L),and one who rejected to insulin therapy, therefore, at last only 35 cases were enrolled in our research.

Pre-pregnancy weight, height, gravidity, and parity history information was extracted from antenatal booklets of their own. Blood examination containing triglyceride (TG), total cholesterol (TC), high-density lipoprotein (HDL), low density lipoprotein (LDL), apolipoprotein A1 (ApoA1), apolipoprotein B (ApoB), and postpartum data were collected by checking their inpatient medical records.

Most of the women were advised to control their own diet for 1 week before admission into hospital, and the poor efficiency compelled them to be hospitalized. After admission to hospital, these patients were followed by a multidisciplinary team consisting of obstetricians, endocrinologists, registered dietitians, and nurse specialists. Supervised low-fat diets with <20% of calories from fat were instituted in conjunction with an intravenous insulin (6–8 U) and 5% dextrose infusion 500 ml three times per day. Within a few days of admission, their plasma triglyceride for about 5–7 days and blood glucose level four times per day were tested, and a final blood examination at discharge was performed. The primary concentration of TGs and TCs was collected from the first blood tests when they registered at the hospital at the gestation of 11–12 weeks initially to build their antenatal booklet, and this level of their lipids in this period was regarded as their pre-pregnancy level as well. The gravida, para, pre-pregnancy weight, and whether smoking or drinking alcohol or any history of chronic diseases were self-reported and documented at their booklets.

About this study, there exists some additional remarks. A few of GDM women enrolled did receive some insulin injection treatments and diet control and exercise for their GDM. About those who were complicated with SCH, most of them were taking levothyroxine sodium tablets orally. Multiple gestations were not necessarily forbidden, but all these women were singleton pregnancies coincidently. The World Health Organization (WHO) defines low birth weight as a birth weight of less than 2,500 g. Conversely, fetal macrosomia is defined as birth weight >4000 g. In this study, we adopted criteria of WHO about birth weight.

### Laboratory Tests

Venous blood samples were collected in the morning after an overnight fast. Serum TGs were measured using the enzymatic colorimetric GPO-PAP method (Siements Healthcare diagnostics Inc), whereas the serum cholesterol level was calculated by the enzymatic endpoint (CHOD-PAP) method on an automatic analyzer (ADVIA Chemistry XPT). Serum low-density lipoprotein-cholesterol (LDL-C) and HDL-C levels were directly measured (dLDL-C) using the homogeneous enzymatic colorimetric assay (Siements Healthcare diagnostics Inc) on an automatic analyzer (ADVIA Chemistry XPT). ApoA1 and ApoB were quantified by routine immuno-turbidimetry methods on an automatic analyzer (ADVIA Chemistry XPT).

### Statistical Methods

SPSS version 25.0 was performed to analyze the whole data. BMI was calculated by dividing pre-pregnancy weight in kilograms by the square of height in meters. A decline of the proportion for TG or TC is equivalent to (TG before treatment − TG after treatment)/TG before treatment. The categorical variables were described by frequency and percentage distribution; the quantitative variables were first tested for normal distribution, and then, they were described with the mean ± standard deviation (x ± s). For continuous variables, a paired-sample *t* test was carried out on each variable to make a comparison between before and after treatment. A comparison of lipid levels between group GDM and non-GDM was performed by independent sample *t* test. A *p* value of 0.05 was considered statistically significant.

## Results

### Notes

1) Age in years, kg, cm, kg/m^2^, g, and days are the units of age, weight, height, BMI, review intervals, and mmol/for TC, TG, LDL, HDL, and baby weight, while g/l is for ApoA1 and ApoB.
2) Gestational week is the time point when this woman was admitted into hospital for hypertriglyceridemia.
3) TG0 and TC0 represent serum triglyceride and total cholesterol before admission into hospital and so did the remaining blood tests. TG1 and TC1 delegate plasma concentration of TG and total cholesterol after intervention of insulin. TG2 and TC2 mean the review of serum triglyceride and total cholesterol after discharge.
4) TG0-TG1 denotes the difference value of serum triglyceride before and after treatment and the same goes for others.
5) GWG means gestational weight gain during the whole pregnancy.
6) GDM means gestational diabetes. SCH means sub-clinical hypothyroidism.

As presented in Table 1, the average age of these people is 31.69 years old, and the median value of their BMI is 24.32 kg/m^2^, which reaches to the edge of overweight according to the Chinese BMI classification standard. There were 48.57% of all the enrolled cases who were diagnosed with GDM, which was not surprising as diabetes can play a role in the development of HTG. The outbreak of gestational lipid disorders occurs mostly around 34 weeks and even later period. Though the minimum GA of admittance was 13. 4 weeks but 29 women went to wards after 30 weeks and four women went to wards between 20 and 30 weeks, only two women were admitted into inpatient hospital between 13 and 20 weeks, which is not presented in the above table. The average pre-pregnancy TGs and TCs of these patients were about 3.96 ± 1.42 and 4.78 ± 1.18 mmol/L separately. The maximum of TG before insulin treatment was up to 64.62 and TC 20.43 mmol/L, which decreased to 17.34 and 4.92 mmol/L after intervention of insulin drip. TG was noticed to fall precipitously by 77% and 12.71% of TG, respectively. Average hospitalization days were approximately 6.71 days while the actual length of hospital days varied from 1 day to 20 days which sometimes was consistent with obedience to the medical order. The difference of TG and TC between pre-treatment and post-treatment was statistically significant (*p* < 0.01), while this difference has not been found in the other laboratory test reports. Of all the patients delivered so far, only three among 28 had premature labor with no less than 36 weeks and their newborn babies, physical condition remain well-being with a 10 min Apgar score achieving at 10. Only one low birth weight newborn was observed of all participants. As presented in Table 2, a total of 30 womens’ TGs and TCs consisting of 15 GDM cases and Nin-GDM cases were extracted from the electronic medical record after discharge until delivery, and we found there showed a significant difference between the initial TGs and TGs after discharge about average 18 days, same with TCs; however, a rebound of TGs and TCs was noticed between the condition on discharge and after discharge. No difference in TGs and TCs or the reduction of theirs was witnessed between groups GDM and non-GDM in these samples.

**TABLE 1:** Characteristics of enrollment of 35 people.

*N*(35)	Minimum	Maximum	mean ± std	*p*
Age	24.00	39.00	31.69 ± 4.50	
Weight	43.00	98.00	63.63 ± 10.69	
Height	153	170	161.11 ± 4.23	
BMI	21.80	26.91	24.32 ± 1.28	
Gravidity	1.00	8.00	2.57 ± 1.44	
Parity	0.00	2.00	0.49 ± 0.61	
GWG	3	34	13.87 ± 6.65	
Smoking	0	0	0	
Alcohol	0	0	0	
GDM (yes/no/percent)	17	18	48.57%	
SCH (yes/no/percent)	8	27	22.86%	
Gestational week	13.43	37.57	32.46 ± 5.91	
Primary TG	2.19	10.65	3.96 ± 1.42	
Primary TC	3.1	8.93	4.78 ± 1.18	
TG0	9.15	64.62	17.76 ± 11.73	
TG1	4.92	17.34	8.94 ± 3.15	
TG0-TG1	−1.28	48.65	8.82 ± 10.51	0.00∗
Decline proportion of TG	−0.08	0.77	0.41 ± 0.22	
TC0	5.41	20.43	9.67 ± 3.91	
TC1	4.18	10.87	6.68 ± 1.53	
TC0-TC1	−1.42	12.71	2.99 ± 3.33	0.00∗
Decline proportion of TC	−0.19	0.71	0.25 ± 0.19	
HDL0	0.72	5.17	1.43 ± 0.80	
HDL1	0.81	2.81	1.34 ± 0.41	
HDL1-HDL0	−0.63	3.96	0.10 ± 0.73	0.53
Decline proportion of HDL	−0.52	0.77	0.03 ± 0.26	
LDL0	0.58	5.23	1.68 ± 1.07	
LDL1	0.74	4.39	1.66 ± 0.75	
LDL0-LDL1	−1.08	1.94	0.01 ± 0.74	0.96
Decline proportion of LDL	−1.34	0.60	0.18 ± 0.47	
ApoA10	0.91	3.50	1.97 ± 0.53	
ApoA11	0.63	3.38	1.78 ± 0.46	
ApoA0-apoA1	−0.97	2.05	0.19 ± 0.48	0.11
Decline proportion of apoA1	−1.07	0.76	0.05 ± 0.29	
ApoB0	0.43	1.82	1.07 ± 0.25	
ApoB1	0.31	1.54	0.97 ± 0.24	
ApoB0-ApoB1	−0.69	1.09	0.11 ± 0.30	0.07
Decline proportion of apoB	−1.60	0.72	0.05 ± 0.36	
Hospital Stay	1	20	6.71 ± 4.64	
Delivery Age	36.00	40.00	37.61 ± 0.96	
Baby weight	2280.00	4000.00	3235.54 ± 344.75	
Apgar point (10 min)	10.00	10.00	10.00	
FBG0	3.8	7.8	4.91 ± 0.90	
FBG1	3.3	5.9	4.52 ± 0.59	
FBG1-FBG0	−1.1	3.4	0.39 ± 0.76	0.01∗
Hypoglycemia	0	0	0	
After Discharge but until delivery(30)				
TG2(30)	3.58	26.27	11.34 ± 5.75	
TC2(30)	4.23	11.9	7.70 ± 2.15	
Review interval(30)	3	74	18.17 ± 16.00	
Review weeks(30)	18	41	35.1 ± 5.20	
TG2-TG0(30)	−7.91	49.16	−6.70 ± 12.50	0.01*
TC2-TG0(30)	−3.59	16.2	−2.34 ± 4.50	0.01*
TG2-TG1(30)	−2.19	13.33	2.21 ± 5.34	0.03*
TG2-TG1(30)	−0.62	5.2	0.85 ± 2.20	0.04*

**TABLE 2:** Lipid levels of the group of GDM and Non-GDM.

Lipid levels of the group of GDM and Non-GDM			
	GDM(17)	Non-GDM(18)	*p*
TG0	16.68 ± 8.68	18.79 ± 14.21	0.60
TC0	9.63 ± 3.52	9.71 ± 4.35	0.96
TG1	8.70 ± 2.05	9.17 ± 4.00	0.66
TC1	6.67 ± 1.34	6.69 ± 1.72	0.98
TG2(15)	11.38 ± 5.87	11.30 ± 5.84	0.97
TC2(15)	8.18 ± 2.26	7.22 ± 2.00	0.23
TG0-TG1	7.99 ± 8.38	9.62 ± 12.40	0.65
TC0-TC1	2.96 ± 3.14	3.01 ± 3.60	0.96
TG0-TG2(15)	5.84 ± 11.70	8.10 ± 13.60	0.63
TC0-TC2(15)	1.94 ± 4.14	2.74 ± 4.93	0.63
TG1-TG2(15)	(−)1.67 ± 4.62	(−)2.75 ± 6.10	0.59
TC1-TC2(15)	(−)1.32 ± 2.06	(−)0.38 ± 2.31	0.25
FBG0	4.95 ± 0.74	4.88 ± 1.05	0.82
FBG1	4.66 ± 0.73	4.39 ± 0.38	0.20

## Discussion

### Pathophysiology of Hypertriglyceridemia in Pregnancy

In order to meet the needs of fetus growth, with increasing intake of sufficient food and elevated secretion of endogenic hormones, including estrogen and human placental lactogen, lipogenesis and hepatic VLDL (very low-density lipoprotein) synthesis are enhanced, and hepatic lipase activity is suppressed. In consequence, levels of plasma concentration of TG and TC are detected higher. Insulin resistance caused by prolactin located in the human placenta introduces recession of the LPL activity and improved lipolysis of the adipose tissue. It follows that the substrates of TG synthesis beef up as free fatty acid rises. As a result, the levels of TG, TC, HDL, and LDL increased significantly with usually no more than three times of non-pregnant levels’ as pregnancy progressed which is called “physiologically hyperlipidemia” (Goldberg and Hegele, 2012; Emet et al., 2013).

In women with abnormal lipoprotein metabolism, these changes lead to severe HTG and may precipitate pancreatitis. GHTG is defined as the fasting plasma triglyceride level above the age-adjusted 95th percentile for the non-pregnant population, while, at the same time, severe HTG implicates triglyceride (TG) ranging from 11.3 to 22.5 mmol/L (1000–1999 mg/dl) and being defined as extremely severe hypertriglyceridemia when the TG of patients is above 22.5 mmol/L (1999 mg/dl).

In our study, there were 24 cases whose TG was more than 11.2 mmol/L, which accounts for above 2/3 of total samples, indicating most of them were enough to be defined as severe HTG.

### Causes and Risk Factors of GHTG

The exact pathogenesis behind GHTG has not yet been fully understood and still remains to be investigated further. Nonetheless, previous studies have demonstrated that HTG can be attributed to overproduction of VLDL, decreased LDL activity and impaired hepatic clearance, genetic defects in apoA-1 alone or in alliance with apoC-III or apoA-IV, or a combination of these. Diet, insulin resistance, reduction of the LDL receptor, and medications can all come into play in this process (Berglund et al., 2012; McCracken et al., 2018).

Risk factors of this hypertriglyceridemia condition includes being overweight, obesity, pre-pregnancy lipid levels, diabetes, inactivity physically, a diet rich in fats, smoking, alcohol consumption, and so on (Mozaffarian et al., 2016). Though these women were not conducted with genetic HTG to further explicit the underlying reasons, there did exist a certain number of risk factors. The average pre-pregnancy BMI of our patients was about 24.32kg/m^2^, which was a situation of being overweight, a risk factor to develop HTG, and this condition was aggravated by insufficient activity in pregnancy. A total of 48.57% of the whole cases were diagnosed with GDM, which, especially poorly controlled diabetes, according to the related literature, is a contributing factor to exacerbate the worse condition of HTG. The average pre-pregnancy TGs and TCs of these patients were about 3.96 ± 1.42 and 4.78 ± 1.18 mmol/L, separately, which exceeds the normal lipid level of non-pregnant women and could be taken as another risk factor. In total, 22.86% of these samples were diagnosed with hypothyroidism or sub-hypothyroidism, which also subsidize the progress of HTG based on the former research studies (Ritter et al., 2020).

### Treatments

In accordance with previous studies conducted in non-pregnant women (Ladizinski and Lee, 2013; Soliman, 2021), our research indicates that insulin drip can reduce the level of TGs and TCs effectively and safely and might become a shining star in the treatment of HTG in this sensitive period. By stimulating lipoprotein lipase (LPL) synthesis and activity, which gives a subsidy to the degradation of VLDL chylomicron to glycerol and free fatty acids, and inhibiting hormone-sensitive lipase in adipocytes, the key enzyme for decomposing adipocytes’ TGs into free fatty acids, insulin reduces the serum triglyceride level rapidly (Ozcelik et al., 2019). The results of our study demonstrated that an average decline of TGs is around 8.82 mmol/L on the condition of the mean length of hospital stay of 6.71 days, while the peak of discrimination of TGs before and after treatment is 48.65 mmol/L. Actually, these data came from a lady whose primary TG and TC was 64 and 20.16 mmol/L, respectively, who accepted insulin therapy about 18 days with a low-fat diet, and her TG returned the level of 15.97 mmol/L while her TC came back to 7.45 mmol/L. Regarding outcomes of mothers and fetus, no cases of hypoglycemia were found in mothers, and the reasons lied in that our intravenous insulin therapy was combined with glucose solution, and our patients were allowed to have low-fat dietary rather than keep fasting though it was demonstrated that a combination of IV insulin and fasting seems more effective by lowering the TG level by about 87% in 24 h (Thuzar et al., 2014). Unfortunately, being in fasting to reduce the TG concentrations cannot be recommended in our study because an adequate and balanced diet is a must in pregnancy not only to sustain the health of the gravida but also for fetal physical and mental growth as well, not to mention there were almost half GDM cases of all participants. Only one low birth weight and one macrosomia were found in the whole newborns with Apgar points of 10, which were acceptable and understandable providing the fact that the two mothers were both diagnosed with GDM. Even after 18 days of insulin drip, the efficiency of insulin was still obvious compared with the original levels of lipids, but it needs to be investigated in the future about how to keep the efficiency stable and constant since those blood tests were noticed to rebound slightly compared with conditions on discharge.

## Conclusion

In brief, this research demonstrates that use of insulin in the managements of GHTG is safe and efficient, and insulin may become a mainstream in the near future to mitigate serum TG and TC levels. Our study is a retrospective research with a small number of samples; however, to the best of our knowledge, use of insulin in the management of GHTG is still limited in case reports, (Mikhail et al., 2005; Ayyavoo et al., 2018; Lee and Kim, 2020), and this is the first clinical trial of application of insulin into GHTG in China, and it represents the largest cohort in the whole world reported so far of patients with HTG dealt with insulin.

In cases of severe HTG in pregnancy, rapid reduction of TG levels matters a lot for successful management of the whole process due to the high chance to develop gestational pancreatitis which is able to pose a threat to the life of both mother’s and fetus’. Our research illustrates that intravenous insulin infusions are a wonderful option in the management of severe HTG in pregnancy with no other therapies which are safe enough.

Allowing for the scarcity of the literature and absence of consensus around management of HTG in pregnancy, we sincerely hope that this article will form a basis for carrying out larger prospective studies to optimize outcomes of both the fetus and mothers in the immediate management of HTG, especially in group of extreme HTG. Future studies are required to delineate ways on how to keep the efficiency of insulin for HTG constant and the optimal timing for induction and delivery, especially in women whom triglyceride levels show a steep upward trend in the third trimester. Strong consideration should be given toward the effects of longer term insulin therapy such as multiple daily injection on GHTG patients.

## Data Availability

The original contributions presented in the study are included in the article/Supplementary Material, further inquiries can be directed to the corresponding author.
