# Corrigendum: Insulin treatment of hypertriglyceridemia during pregnancy

**DOI:** 10.3389/fphar.2022.972477

**Published:** 2022-09-23

**Authors:** De-cui Cheng, Yao Su, Feifei Li, Xianming Xu

**Affiliations:** Department of Obstetrics and Gynecology, Shanghai General Hospital, Shanghai, China

**Keywords:** insulin, hypertriacylglycerolemia, pregnancy, hypercholesterolemia, fetus

In the published article, there was an error in the Affiliation. Instead of “Department of Obstetrics and Gynecology, Jiao Tong University School of Medicine, Shanghai, China,” it should be “Department of Obstetrics and Gynecology, Shanghai General Hospital, Shanghai, China.”

Additionally, a correction has been made to **Methods** section, paragraph 1. The last sentence previously stated “This research was registered with the Chinese Clinical Trial Registry (ChiCTR2000036575).” However the correct sentence is “This research was funded by the Chinese Clinical Trial Registry (ChiCTR2000036575).” The **Conflict of Interest** statement has also been updated to reflect this.

The authors apologize for these errors and state that they do not change the scientific conclusions of the article in any way. The original article has been updated.

